# Identifying Acute Coronary Syndrome Patients Approaching End-of-Life

**DOI:** 10.1371/journal.pone.0035536

**Published:** 2012-04-18

**Authors:** Stephen Fenning, Rebecca Woolcock, Kristin Haga, Javaid Iqbal, Keith A. Fox, Scott A. Murray, Martin A. Denvir

**Affiliations:** 1 Edinburgh Heart Centre, Royal Infirmary of Edinburgh, Little France Crescent, Edinburgh, United Kingdom; 2 Department of Cardiovascular Science, University of Sheffield, Sheffield, United Kingdom; 3 Primary Palliative Care Research Group, Centre for Population Health Sciences, University of Edinburgh, Edinburgh, United Kingdom; S.G.Battista Hospital, Italy

## Abstract

**Background:**

Acute coronary syndrome (ACS) is common in patients approaching the end-of-life (EoL), but these patients rarely receive palliative care. We compared the utility of a palliative care prognostic tool (Gold Standards Framework (GSF)) and the Global Registry of Acute Coronary Events (GRACE) score, to help identify patients approaching EoL.

**Methods and Findings:**

172 unselected consecutive patients with confirmed ACS admitted over an eight-week period were assessed using prognostic tools and followed up for 12 months. GSF criteria identified 40 (23%) patients suitable for EoL care while GRACE identified 32 (19%) patients with ≥10% risk of death within 6 months. Patients meeting GSF criteria were older (p = 0.006), had more comorbidities (1.6±0.7 vs. 1.2±0.9, p = 0.007), more frequent hospitalisations before (p = 0.001) and after (0.0001) their index admission, and were more likely to die during follow-up (GSF+ 20% vs GSF- 7%, p = 0.03). GRACE score was predictive of 12-month mortality (C-statistic 0.75) and this was improved by the addition of previous hospital admissions and previous history of stroke (C-statistic 0.88).

**Conclusions:**

This study has highlighted a potentially large number of ACS patients eligible for EoL care. GSF or GRACE could be used in the hospital setting to help identify these patients. GSF identifies ACS patients with more comorbidity and at increased risk of hospital readmission.

## Introduction

The importance of well-coordinated supportive care at end of life (EoL) is increasingly recognised as more people survive into old age with multiple chronic long term conditions. While significant improvements have been made in palliative care for cancer patients, this is not so for patients with heart disease. In the last 10 years there have been many calls for better EoL care for people with advanced heart failure [1]–[5] with little or no attention to other chronic heart conditions where very similar issues apply such as advanced valvular heart disease, congenital heart disease and coronary heart disease syndromes. Despite calls for better EoL care there continues to be a lack of research examining ways in which it could be integrated into normal clinical care pathways especially in the acute hospital setting. We know that cardiac patients generally receive limited information about their condition and have poor access to EoL care services [6], [7]. Furthermore, EoL care is often poorly co-ordinated, with inadequate communication and cohesion between primary care, cardiologists and palliative care specialists [8], [9]. These problems have been attributed to clinicians being in a state of “prognostic paralysis”, uncertain about the illness trajectory of heart disease and thus uncertain about when to initiate EoL care planning [10].

Acute coronary syndrome is a common precursor to, and cause of, death, particularly in the elderly. Indeed, the use of high sensitivity troponin assays has resulted in many patients being redirected from more generalist medical care to acute cardiology services where they often receive excellent cardiac care but rarely benefit from additional multi-dimensional support which they might need [11]. A recent study found that nearly 25% of all ACS patients over the age of 75 years were significantly frail with increased short term mortality [12].

Focusing clinicians on the task of identifying patients with a prognosis of less than a year is increasingly recognised as useful trigger to raise awareness of the need for EoL care, as well as cardiac care, in the acute hospital setting. There is a clear need to explore, develop and test the utility of prognostic tools that could assist clinicians to recognise such patients particularly in the acute hospital setting.

The Gold Standards Framework (GSF) was initially developed for cancer patients and more recently was broadened to include chronic neurological, lung, kidney and heart disease [13]. It aims to identify people approaching the end-of-life using clinical and functional criteria that were derived and agreed using expert clinical opinion and consensus (see Table 1). Many of these criteria are subjective and have never been validated in a prospective cohort of patients with heart disease. The GRACE score [14], in contrast, is based on clinical data from over 100,000 patients presenting to hospital with ACS and has been extensively validated in numerous prospective studies [15]–[18].

**Table 1:** Gold Standards Framework criteria.

**General Criteria of End-stage illness (at least one of these)**
• Weight loss >10% in last 6 months
• General physical decline
• Serum albumin <25 g/l
• Reducing performance status (Karnofsky score <50%)
**Heart Disease specific criteria (at least 2 of these)**
• The “Surprise Question” (to be asked of a health care provider familiar with the patient): “*Would you be surprised if this patient died in the next 6 to 12 months?*”
• New York Heart Association (NYHA)– Stage III or IV heart failure
• Repeated hospital admissions within the last year
• Difficult physical or psychological symptoms despite optimised tolerated therapy

This study aimed to assess two specific issues. Firstly, to compare the utility of the GSF (GSF) criteria and the GRACE score in identifying patients discharged from hospital following an admission with acute coronary syndrome that may be approaching the last year of life. Secondly, to assess the prevalence and clinical characteristics of ACS patients who meet criteria for EoL care.

## Methods

### Subjects

Consecutive unselected ACS patients from one health authority region admitted to a large urban-based hospital cardiology unit over a two month period were included. All patients had a confirmed diagnosis of acute coronary syndrome according to national guidelines [19] including at least 2 of the following : typical cardiac chest pain, a rise in plasma markers of cardiac injury and electrocardiographic evidence of myocardial ischaemia or infarction. Since we aimed to focus on EoL needs following discharge, patients admitted with ACS who died in hospital were excluded from analysis.

### Data collection and interviews with medical staff

Data were collected from patient records prior to discharge on specifically designed audit forms by research staff. These forms included data fields for the various GSF criteria, clinical and biochemical parameters required to calculate the GRACE score. Members of the medical team caring for the patient during their in-patient stay had brief training on the content of the GSF and were interviewed to obtain data for the GSF criteria including the Karnofsky performance score [20], assessment of recent general physical decline, presence of ongoing difficult symptoms. These medical staff also provided an answer to the “surprise question” (see Table 1). According to the GSF criteria, patients who score positively on one general criterion and two heart-disease specific criteria would benefit from a review for EoL care needs.

### Clinical follow-up

Follow-up at 6 and 12 months was undertaken using a hospital-based electronic patient record system and all-cause mortality events were checked using a national central health index database.

### Ethical considerations

All data were collected and collated anonymously and stored according to the Data Protection Act, United Kingdom (1998). The study was granted audit status by South East Scotland Research Ethics service as part of a clinical implementation project assessing the use of the GSF in a hospital setting. No patients were interviewed and there was no specific patient intervention.

### Statistical analysis and data handling

The GRACE score was calculated for each patient using a web-based calculator available from the GRACE website as a raw score (range 40–220). The raw score was used to define low, intermediate and high risk tertiles and is also presented as a percentage 6 month mortality risk based on the discharge to 6 month follow up algorithm (see http://www.outcomes-umassmed.org/grace/).

Data are expressed as mean± standard deviation unless otherwise stated. Analysis was carried out using Student's t-test for continuous data and Chi square and Fisher's Exact tests for categorical data. Variables with significant trend (P<0.1) were entered in Cox proportional-hazards regression survival model to identify factors independently affecting mortality. Statistical significance was accepted at the 5% level. For parameters demonstrating significance in the multivariate analysis, survival curves were computed using the Kaplan–Meier method. ROC curves were plotted using predicted vs. actual mortality with GRACE risk alone and combination of GRACE with stroke and admissions. PASW Statistics 18 software (IBM corporation, New York, USA) was used for analysis.

## Results

### Patient characteristics

172 patients were included in the study (Table 2). In summary, 60% were male with mean age 66 years. At presentation, the mean heart rate was 76/minute, mean systolic blood pressure 140 mmHg and mean creatinine 103 umol/l. Troponin was elevated in 70%, ST segment deviation on the electrocardiogram was present in 56% and 26% had evidence of heart failure during admission. Two or more additional comorbidities were present in 37% (61) including for example diabetes, arthritis, chronic lung disease, stroke, or chronic kidney disease. The majority of patients were treated appropriately according to national guidelines with evidence based drugs with no significant differences in use of invasive strategy in those that survived compared with those that died within 12 months (Table 2).

**Table 2:** Baseline patient characteristics and alive versus dead by 12 months comparison.

	All patients (n = 172)	Alive (n = 155)	Dead (n = 17)	ttest/Chi/Fisher
Age	66±14	64±14	79±8	<0.00001
Gender (Males,%)	61	62	53	0.64
**GRACE score criteria** (mean±SD)				
Heart rate (min^−1^)	76.2±19.3	76.2±19.0	76.4±22.5	0.96
Systolic blood pressure (mmHg)	140.8±27.0	140.7±27.2	142.2±25.0	0.83
Creatinine (µmol/l)	103.1±52.1	101.7±53.2	116.3±40.4	0.29
Killip Class (1–4)	1.3±0.5	1.3±0.5	1.4±0.6	0.27
Cardiac arrest (%)	4	4	0	
ST deviation (%)	56	56	53	0.50
Elevated troponin (%)	65	63	77	0.21
Probability in-hospital death (%)	3.4±5.3	2.6±3.0	9.0±11.7	<0.0001
Probability in-hospital death/MI (%)	12.3±6.5	11.9±6.3	15.5±7.6	0.08
Probability 6 month death (%)	6.0±6.7	5.5±6.4	10.9±7.5	<0.001
Probability 6 month death/MI (%)	17.6±9.1	12.1±6.6	23±13.2	<0.001
**GSF criteria** (mean±SD)				
Albumin (g/dl)	41.0±4.1	41.1±4.0	40.2±4.6	0.34
Surprise question “No” (%)	22	21	35	0.14
GSF-End stage illness (n)	0.7±0.8	0.6±0.8	1.1±0.9	0.05
GSF-Heart Disease (n)	1.0±1.2	1.0±1.1	1.6±1.3	0.03
GSF-Combined criteria (n,%)	23	21	20	0.02
12 months prior to admission (n)	0.3±0.6	0.3±0.6	0.6±0.9	0.02
12 months following admission (n)	1.2±1.7	1.0±1.4	2.8±2.8	<0.0001
**Co-morbidity**				
Total co-morbidities (n, mean±SD))	1.3±0.9	1.3±0.9	1.6±0.8	0.08
Previous Stroke (%)	8.7	7.7	17.6	0.17
Diabetes (%)	23	23	18	0.43
COPD (%)	6	5	24	0.01
CKD (%)	8	6	24	0.04
Other comorbidity (%)	68	67	76	0.32
**In-patient/discharge treatment (%)**				
Aspirin	94.2	94.8	88.2	0.25
Clopidogrel	93.6	93.5	94.1	0.33
Heparin	98.8	99.4	94.1	0.20
Statin	79.7	80.6	70.6	0.51
ACE inhibitor	65.1	65.2	64.7	0.57
Beta blocker	58.7	59.4	52.9	0.61
Angiography	69.8	70.3	64.7	0.59
Percutaneous intervention	48.8	47.7	58.8	0.45

P values for alive versus dead comparison, GSF – Gold Standards Framework, SD standard deviation, ST – electrocardiogram ST segment, COPD – chronic obstructive pulmonary disease, CKD – chronic kidney disease.

### Identifying End of Life using GSF

Defining a positive GSF status as one general criterion plus two heart disease criteria identified 40 (23%) patients as approaching EoL. During follow-up, GSF positive patients were more likely to die than GSF negative patients (20% vs 7%, p = 0.03). GSF positive patients had a significantly greater number of additional comorbidities (1.6±0.7 vs 1.2±0.9, p = 0.007) and were more likely to have recurrent hospital admissions both before and after their index admission for this study (Table 2).

Using only two heart disease criteria to define EoL would have been identified 47 patients (27%) as GSF positive. Using the GSF ‘surprise’ question as the sole prognostic indicator would have identified 38 (22%) patients likely to die within the next 6 to 12 months. General physical decline was identified in 45 patients (26%), difficult ongoing symptoms in 31 (18%) and a low Karnofsky performance score in 24 (14%). Weight loss, as defined in the GSF, was a difficult to assess mainly because most patients were unaware of their previous and current weight and so this could not be accurately assessed across the whole cohort. There were no patients with albumin levels lower than 25 g/l in whole cohort.

### Identifying End of Life using GRACE

The GRACE risk score was not significantly higher in patients who were GSF positive using one general and two heart disease criteria (Table 3). In this consecutive cohort of unselected patients, GRACE identified 72(42%) patients with a ≥5%, 32 (19%) patients with a ≥10% and 8 (5%) patients with a ≥20% risk of death within 6 months from discharge. Patients with a 6 month mortality risk of ≥10% had significantly more co-morbidities (1.5+0.8 vs 1.2+0.9, P = 0.014) and a higher proportion were GSF positive (50% vs 17%, p = .0.003) compared with patients with a GRACE risk of less than 10%. Combining GSF positive patients with an estimated GRACE risk of death of 10% or more would have identified 16 (10%) patients as suitable for EoL care.

**Table 3:** Clinical characteristics and outcomes of Gold standards Framework (GSF) positive (defined as meeting 1 general criterion and 2 heart disease criteria) versus Gold Standards Framework negative patients.

	GSF positive	GSF negative	P (ttest/Fisher)
	(n = 40)	(n = 132)	
Age (years)	71±12	64±14	0.006
Male (%)	62.5	60.6	0.76
Death by 12 months (%(n))	20.0 (8)	6.8 (9)	0.03
Additional comorbidities (n)	1.7±0.7	1.2±0.9	0.004
**GRACE criteria**			
Heart rate (min^−1^)	81.4±21.7	74.7±18.3	0.10
Systolic BP (mmHg)	140±29	141±27	0.7
Creatinine (µmol/l)	107±32	102±57	0.6
ECG ST deviation (%)	47.5	4.5	0.001
Elevated troponin (%)	70.0	58.3	0.25
Killip class (1–4)	1.5±0.6	1.2±0.5	0.0005
GRACE 6 month death risk (%)	6.1±7.0	6.0±6.6	0.92
**Admissions (n)**			
12 months prior to admission	0.8±0.8	0.2±0.5	0.001
12 months following admission	2.4±2.6	0.8±1.2	0.0001

### Follow-up

**Figure 1:**
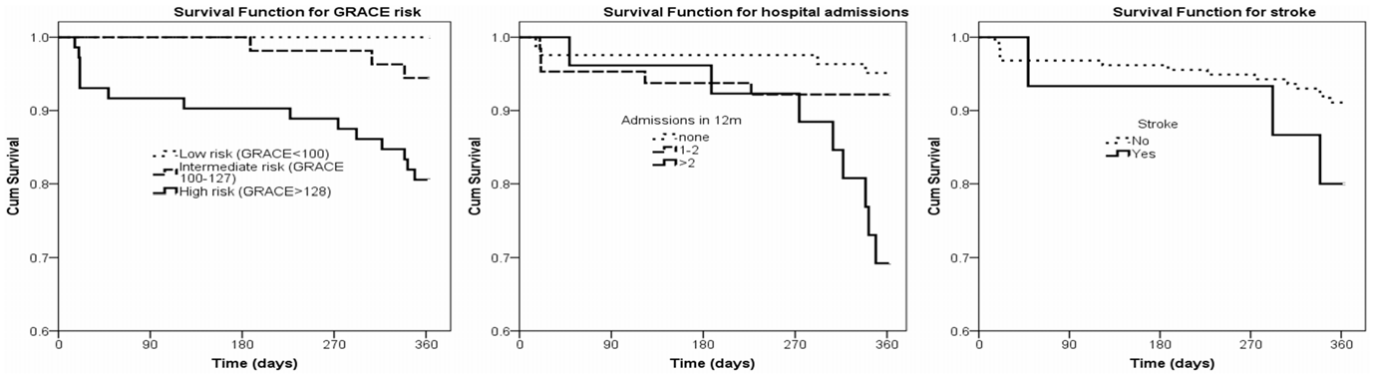
Kaplan-Meier survival curves for factors independently predicting all-cause mortality.

At 6 months, 6 patients had died while a further 11 died by 12 months. Patients that died by 12 months follow-up (n = 17) had a significantly higher discharge raw GRACE score (146±20 vs 115±32, p = 0.0001) and consequently had a significantly higher estimated mortality risk (10.9±7.5% vs 5.5±6.4%, p = 0.001). Based on tertiles, GRACE score at discharge was highly predictive of death (Table 4) clearly distinguishing between those at low, medium and high risk of death by 12 months (Figure 1). Patients that died were significantly more likely to have had non-elective hospital admission during the year prior to their index admission (p = 0.02) and were also more likely to have readmission during 12 month follow-up (p = <0.0001, Table 2). GSF positive patients had a significantly greater number of readmissions during follow-up compared with GSF negative patients (2.4±2.6 vs 0.8±1.2, p<0.0001). After correction for other factors, the GRACE risk score (tertiles), previous stroke and 3 or more previous non-elective hospital admissions within the last year were independently predictive of death (Table 4 and Figure 1).

**Table 4:** Univariate and multivariate analysis of factors predicting all-cause mortality.

	Uni-variate^1^	Multivariate^2^
Gender	0.5	N/A
GRACE score Tertiles	0.001	0.04
**GSF General Criteria**		
General Physical decline	0.3	N/A
Albumin (<25)	N/A^3^	N/A
Karnofsky score	0.87	N/A
**GSF Heart Criteria**		
Difficult symptoms	0.4	N/A
Surprise question	0.06	0.5
Repeated hospital admissions	0.001	0.001
**Co-morbidities**		
Total co-morbidities (n)	0.3	N/A
Stroke	0.1	0.04
Diabetes	0.6	N/A
COPD	0.002	0.1
CKD	0.01	N/A^4^

1 p value from Kaplan Meier log-rank test.

2 p value from Cox-regression analysis.

3 No patient with less than 25 g/dl albumin.

4 CKD is not independent risk factor as creatinine level included in GRACE score.

### Sensitivity and specificity of GRACE and GSF for 12 month all-cause mortality

**Figure 2:**
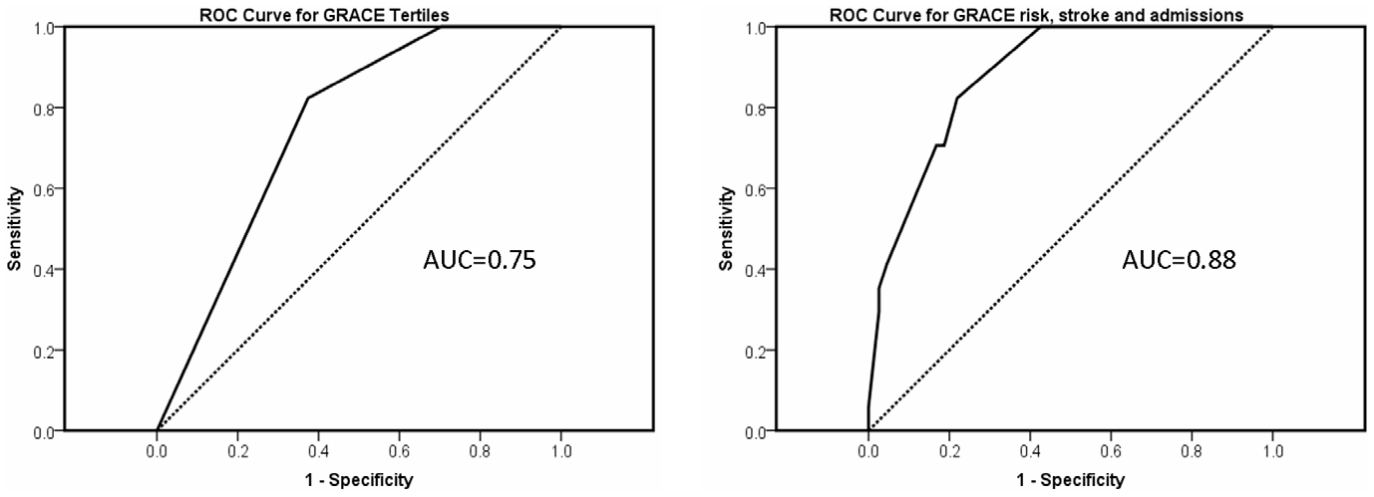
ROC curve for predicted mortality from GRACE risk alone and combination of GRACE risk with previous stroke and previous hospital admissions.

The sensitivity and specificity of GRACE and GSF in predicting 12 month mortality are summarised in Table 5. Both approaches provided a reasonable specificity although this was higher for the GRACE score. Using a cut-off of 20% for GRACE did not improve the sensitivity or specificity in predicting death. The predictive value of GRACE, however, improved with addition of stroke comorbidity and hospital admissions (Figure 2) with an increase in the area under the ROC curve from 0.75 to 0.88.

**Table 5:** Sensitivity and specificity of prognostic scores.

	Sensitivity (%)	Specificity (%)	NPV (%)	PPV (%)
GSF (1 general, 2 heart disease)	47	79	93	20
GRACE (upper tertile)	82	75	98	26
GRACE (upper tertile)+GSF	78	89	97	44
“Surprise question”	35	79	92	16

NPV – negative predictive value, PPV – positive predictive value, GSF – Gold Standards Framework criteria positive, GRACE – Global Registry of Acute Coronary Events.

## Discussion

This unique study has highlighted a potentially large number of patients presenting to hospital with ACS who could benefit from enhanced supportive care as they approach end of life. GSF and GRACE, used independently, would identify around 20% of an unselected cohort of ACS patients presenting to a large urban hospital as suitable for EoL care according to agreed criteria developed for cancer patients.

In the United Kingdom, these patients could be included on palliative care registers held by community physicians who then ensure that patients receive an assessment of their care-needs, develop an advanced care plan which includes detailed discussion with the patient of future aspects of management and resuscitation status. This type of care planning, sometimes referred to as advanced care planning, is associated with reduced readmission to hospital [21]–[23]. However, to date, there are little or no data available to assess the resources that would be required to deliver this to a high standard for all eligible patients. Our study is the first that we are aware of to highlight this in a disease specific way.

The number of patients referred for EoL care could be more than halved if the GRACE and GSF tools were used in combination. However, this combined approach would miss a significant number of patients nearing EoL who might benefit from this care. The negative predictive power would be strong however and so if a patient was GSF negative and had an estimated mortality risk of less than 5% at discharge then they would be very unlikely to die or be readmitted within the following 12 months. Furthermore, patients identified as requiring EoL care by either GRACE or GSF in our study were older, had more comorbidity, were more likely to be readmitted during follow up and had higher mortality than those who did not meet these criteria. This would suggest that in addition to identifying risk-of-death in the setting of ACS, these tools are also able to identify patients with greater needs and who are at risk of hospital readmission. Our findings are similar to a recent study assessing frailty in elderly ACS patients [12]. This is not unexpected since the GSF criteria used in our study contain measures of functional status similar to those used in frailty assessment scores. However, we have gone further by raising the issue that these patients could benefit from an advanced care plan combined with extra-supportive care in the community and indeed some may benefit from review by a palliative care specialist.

Acute coronary syndrome in elderly patients, as defined by cardiac symptoms with elevation of plasma biomarkers, has become increasingly common with the introduction of high sensitivity troponin assays [24]. Cardiac injury may occur with other chronic conditions associated with ageing such as chronic kidney disease [25], chronic lung disease [26] and pulmonary embolic disease [27], all of which are common conditions in elderly people admitted to hospital. In these clinical settings clinicians should perhaps be acknowledging ACS as a syndrome associated with the end of life. This concept does not in any way imply that such patients should not receive optimal evidence-based therapies during their in-patient stay, this should be assured. However, the clear challenge is to use an admission with ACS in an elderly patient with multiple co-morbidities as an opportunity to carefully consider the global functional status of the patient. Thereafter, expert clinical judgement combined with appropriate clinical data should be used to increasingly provide extra supportive care while continuing to provide disease modifying treatment, a concept now realised in cancer-care. The findings of this study have clearly highlighted the need for such a change in approach.

Our study indicated that many ACS patients with the increased care needs could be identified by asking the ‘surprise’ question although further assessment using GSF criteria or GRACE score would be necessary. Patients highlighted by medical staff as at risk of dying within 6 to 12 months were older, had more co-morbidity and scored lower on functional status. In fact, our findings suggest that asking hospital doctors to assess patients during a relatively short in-patient stay using the ‘surprise’ question would have strong negative predictive accuracy in detecting ACS patients who might be approaching EoL. Therefore, it seems appropriate that, as a screening tool, doctors should be encouraged to ask themselves the ‘surprise’ question when considering the illness trajectory of their patients.

### Limitations

There are several limitations to this study. Firstly, we studied a relatively small number of patients, although with a fairly detailed interview with the patient's caring physician, and so further research is warranted to examine many of the complex issues associated with identification of patients presenting in the acute hospital setting who could benefit from EoL care. The GRACE model does not incorporate non-cardiac co-morbidities and in this setting these may play a more important role in management of the patient. By interviewing different doctors over the 8 week period of the study this may also have introduced the potential for inter-observer variability. However, we did provide the same brief education intervention to all doctors who provided interviews in a way that could be replicated elsewhere. The multivariate analysis is also limited by the small sample size and the relatively small number of mortality events.

In conclusion, this study has highlighted a potentially large number of cardiac patients admitted to hospital with ACS who by various criteria, as defined by GRACE and the GSF, may be approaching end of life. We have also demonstrated that both prognostic tools have excellent rule-out utility and furthermore they appear to identify a group of patients with increased care-needs.

While further research is needed, the time has come for cardiologists and physicians caring for ACS patients to consider, at discharge, the need for extra supportive care in the community for those identified as approaching end of life while not denying them optimal evidence based care.
